# A new method to estimate the histological stage of primary biliary cholangitis

**DOI:** 10.1007/s00330-023-10106-3

**Published:** 2023-08-22

**Authors:** Yuan Zhang, Xing Hu, Jing Chang, Weihua Li, Chunyang Huang, Haiping Zhang, Jianjun Shen, Ning Shang, Fankun Meng

**Affiliations:** 1 Beijing Youan Hospital, Capital Medical University, No 8, Xitoutiao, Youanmenwai, Fengtai District, Beijing, 100069 China; 2 Handan Infectious Disease Hospital, Congtai District, No. 472, Heping Road, Handan, China

**Keywords:** Primary biliary cholangitis, Ultrasound imaging, Liver fibrosis, Inflammation, Cholestasis

## Abstract

**Objective:**

To analyze the diagnostic efficacy of the periportal hypoechoic band (PHB) in the histological stage of patients with primary biliary cholangitis (PBC).

**Methods:**

We prospectively included 77 cases of PBC pathologically or clinically confirmed, and high-frequency ultrasound (HFUS) measurements of the PHB were performed in all included patients. Ludwig staging system of histopathology was used as the gold standard.

**Results:**

The width of the PHB was positively correlated with histological staging (*r* = 0.844, *p* < 0.001). By area under the receiving operating characteristic curve (AUROC), the best cutoff value for PHB for advanced stage (≥ stage 3) was 2.4 mm (AUROC: 0.934; 95%CI: 0.841–0.981) and 0.93 for sensitivity, and 0.91 for specificity, the concordance rates of PHB vs. liver biopsy was 90.3%. The correct rate for early-stage PBC was 87.9% and for the progressive stage was 93.1%. After multi-factor regression analysis, the PHB (OR = 1.331, CI = 1.105–1.603, *p* = 0.003) and total bilirubin (OR = 1.156, CI = 1.041–1.285, *p* = 0.007) were independent influencing factors for progressive PBC.

**Conclusions:**

Measurement of the PHB to assess advanced PBC is a simple and effective method. This method may complement current methods for the histological staging assessment of patients with PBC.

**Registration:**

Clinical trial registration: ChiCTR 2000032053, 2020/04/19.

**Clinical relevance statement:**

The measurement of periportal hypoechoic band (PHB) provides a simple and easy assessment of the degree of disease progression in patients with PBC and provides an important clinical reference in predicting the histological staging of PBC from an ultrasound perspective.

**Key Points:**

*• The PHB is correlated with histological staging in the patient with PBC.*

*• The area under the ROC curves of PHB for detecting advanced stage (≥ stage 3) were 0.934 and 0.93 for sensitivity, and 0.91 for specificity, the concordance rates of PHB vs. liver biopsy was 90.3%. The application of PHB can better assess the advanced PBC.*

*• Measurement of the PHB to assess advanced PBC is a simple and effective method that can significantly reduce the need for liver biopsy.*

## Introduction

Primary biliary cholangitis (PBC) is an autoimmune liver disease characterized by inflammation of the interlobular bile ducts and interstitial bile duct cells. The natural course of disease tends to be progressive. In the absence of proper intervention, a vast majority of patients diagnosed with PBC will succumb to liver failure, necessitating either liver transplantation or resulting in death, within a decade [1]. Patients with early-stage PBC are better treated to reduce inflammation and bile duct hyperplasia around the portal tracts, while treatment for patients with mid to late-stage PBC is mostly focused on symptomatic treatment such as reducing portal hypertension, reducing ascites and preventing bleeding from ruptured esophagogastric varices. Therefore, determining the degree of disease progression in PBC is crucial to treatment and prognosis. While liver biopsy is considered the gold standard for determining liver histological staging, several factors, including the heterogeneity of liver tissue, subjectivity of the observer, and the potential risk of bleeding, must be taken into account, only 20% of patients with PBC are clinically indicated for liver biopsy to clarify the etiology [2]. Although Transient elastography (TE) is recommended for use in the diagnosis and follow-up of PBC disease staging [3], it is not universally available in all health-care settings and is limited by numerous factors such as elevated bilirubin and abdominal wall thickness [4].

As the disease progresses, the number of bile ducts in PBC patients decreases or even disappears, and increased inflammation in the portal area enters the liver parenchyma, the periportal area, and periseptal areas where bile accumulates. Although only bile ducts less than 100 μm in diameter are involved in PBC, the imaging change has been found around the larger portal vein branches. Magnetic resonance imaging (MRI) showed a halo ring sign around the portal vein wall [5], presumably due to periportal fibrosis or hepatocyte depletion, or inflammatory cell infiltration [6]. At present, there was no relevant study on the relationship between ultrasound image changes and histological staging in PBC patients. However, it has been reported in earlier studies that low-reflectivity periportal collar ultrasonographic can be observed around portal veins in patients with PBC, and we speculate that low-reflectivity ultrasonographic was associated with PBC histological staging. The aim of this study was to evaluate the histological staging of PBC using two-dimension ultrasound, a widely used clinical technique, simply, quickly, and non-invasively by measuring the width of the PHB.

## Material and methods

### Patients

This prospective study was approved by the Ethics Committee of Beijing Youan Hospital and informed consent was obtained from all subjects. We prospectively enrolled 77 consecutive patients with PBC who presented at our hospital between February 2019 and March 2022 with confirmed liver histopathology and/or clinical diagnosis and enrolled 56 hepatitis B virus (HBV) patients as controls during the same period, with inclusion criteria meeting the latest guideline criteria [3, 7], respectively. A ultrasound examination was performed within 3 months of liver biopsy. Clinical and anthropometric data were collected at the time of the ultrasound examination. Clinical data collected included: alanine aminotransferase (ALT), aspartate aminotransferase (AST), total bilirubin (TBIL), alkaline phosphatase (ALP), and gamma-glutamyl transferase (GGT). We have recruited a cohort of 8 patients diagnosed with PBC, from January 2023 to March 2023, to assess the consistency of PHB measurement across observers with varying levels of seniority.

### Ultrasonographic measurements

Ultrasound images were acquired and analyzed by two sonographers with more than 10 years of experience in abdominal ultrasonography without knowledge of histological staging as well as clinical aspects. The width of the periportal hypoechoic band (PHB) was measured using a SuperSonic Aixplorer ultrasound diagnostic instrument with a 5–12 MHz transducer. The width of the PHB was measured by selecting the hypoechoic width around the rami inferior segment of the left external lobe of the portal vein, using local magnification and averaging three measurements.

Earlier in the study, to assess inter-observer agreement, two operators performed parallel double-blind ultrasound examinations on 10 patients with PBC who had histological staging of stage 1:1, stage 2:4, stage 3:1, and stage 4:4 subjects respectively. In this study, inconsistent results were resolved by discussion between the two operators. Later in the study, three ultrasound doctors with varying levels of seniority (< 10 years, 10–20 years, and > 30 years of experience, respectively) were tasked with measuring the width of PHB in the aforementioned cohort of 8 PBC patients. The primary aim was to explore potential differences in PHB observation between ultrasound doctors with different years of experience and to determine the level of inter-observer consistency between them.

### Liver histologic examination

The pathological findings were diagnosed by an experienced pathologist without knowledge of the clinical and imaging findings. Liver biopsy specimens are between 1.5 and 2.0 cm in length and have ≥ 8 portal areas. PBC was staged by the Ludwig system and divided into stage 1, stage 2, stage 3, and stage 4 [8], and early stage was defined as stage 1–stage 2, and the progressive stage was defined as stage 3–stage 4. HBV was staged by the METAVIR scoring system and divided into F0, F1, F2, F3, and F4 [9].

H/E stained, Masson stained and CK7 immunohistochemistry sections of PBC and HBV patients were compared under a multiheaded microscope. Due to the difference in diagnostic criteria for pathological staging of the two diseases, to further compare the differences in the degree of portal inflammation and cholestasis around the portal area with the same degree of liver fibrosis, subgroups were made by fibrosis score: group A (fibrosis score 0), group B (fibrosis score 1), and group C (fibrosis score 2–3). The scoring criteria were as follows. Liver fibrosis was scored as follows: 0 = no fibrosis or fibrosis limited to portal tracts; 1 = portal fibrosis without septa; 2 = bridging fibrosis;3 = cirrhosis. Portal inflammation was scored as 0 = none; 1 = interface hepatitis affecting about 10 continuous hepatocytes in one portal tract; 2 = interface hepatitis affecting about 10 continuous hepatocytes in two or more portal tract; 3 = interface hepatitis affecting about 20 continuous hepatocytes in more than half of portal tract. Cholestasis was scored as 0 = no cholestasis; 1 = mild; 2 = moderate; 3 = severe.

### Statistical analysis

SPSS 22.0 statistical, MedCalc (version 15.2.2), and GraphPad Prism (8.0.2) software were applied for data analysis. Enumeration data were expressed as rates. The measurement data were expressed as mean ± standard deviation or median. A t-test or rank-sum test was used for comparison between the means of two groups, and a one-way ANOVA or non-parametric test was used for comparison among the means of multiple groups. The different parameters were analyzed with histological staging using Spearman rank correlation analysis. The intraclass correlation coefficient (ICC) was used to evaluate the inter-observer agreement. Univariate and multivariate regression analysis was used to analyze the risk factors for the advanced-stage patients with PBC, we selected age, sex, BMI, ALT, AST, TBIL, ALP, GGT, and PHB. A *p* value < 0.05 was considered statistically significant. The mean replacement method was applied to handle missing data.

The sensitivity-to-specificity relationship of each noninvasive diagnostic test was assessed with receiver operating characteristic (ROC) curves. The area under the receiver operating characteristic curve (AUROC) and the 95% confidence interval of the AUROC were calculated for the detection of stage ≥ 2, stage ≥ 3, and stage = 4. Cutoff values between histological staging were determined at the maximum sum of sensitivity plus specificity.

## Results

### Study population

**Fig. 1:**
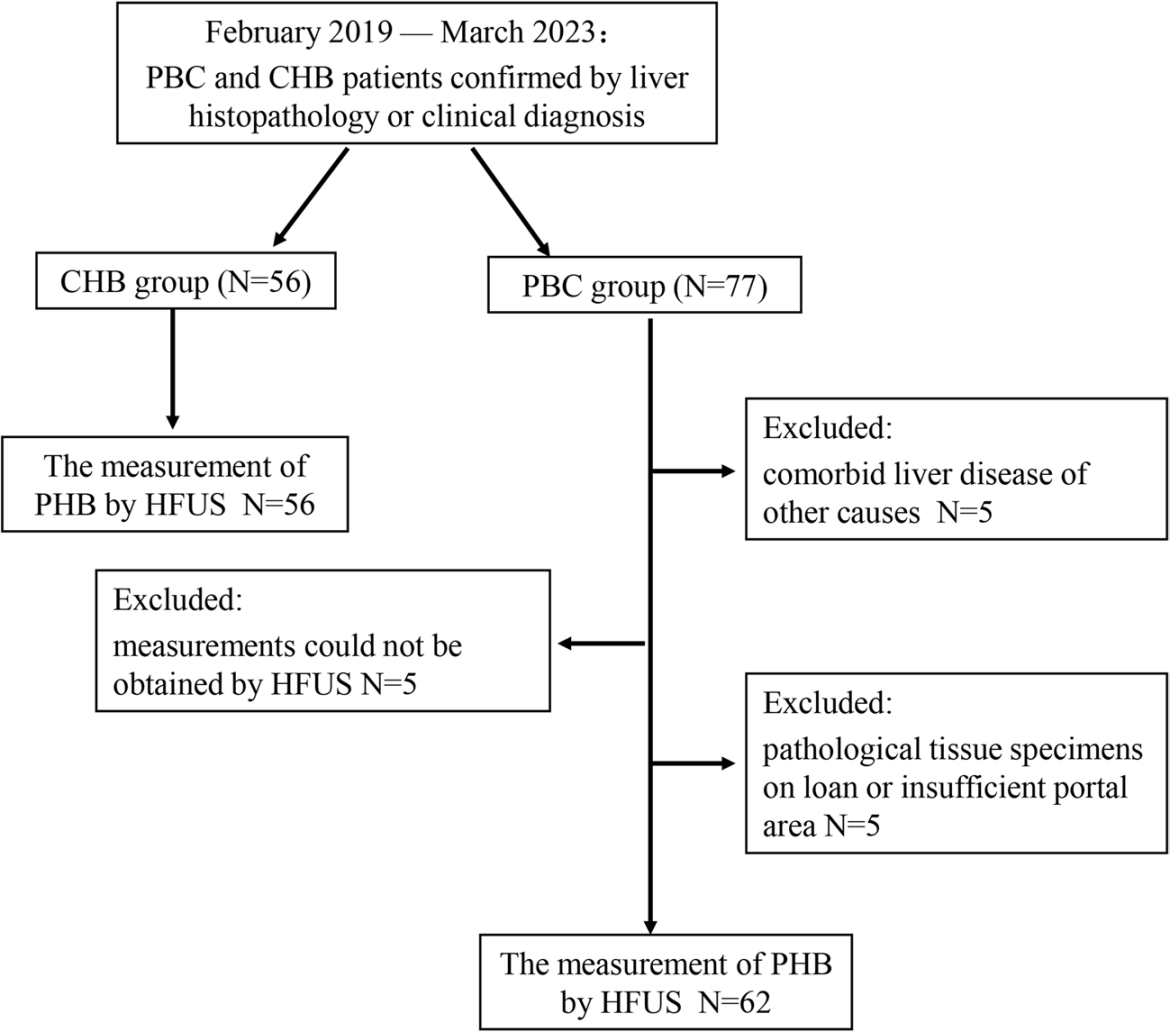
Study flow chart

Seventy-seven PBC patients were prospectively recruited and 15 (19.5%) were excluded for not meeting the inclusion criteria, including 5 patients due to comorbid liver disease of other causes, 5 cases of obesity and abdominal distention where high-frequency ultrasound (HFUS) measurements could not be obtained, and 5 cases of pathological tissue specimens on loan or insufficient portal area. A final total of 62 patients with PBC (6 men and 56 women) were included. Concurrently matched 56 HBV patients (20 men and 36 women). The median age was 50 (16–78). PBC patients including 11 stage 1, 22 stage 2, 10 stage 3, and 19 stage 4, and HBV patients including 17 F1, 13 F2, 12 F3, and 14 F4 (Fig. 1). Eighteen patients with PBC stage 4 and 14 patients with HBV F4 were clinically confirmed and did not undergo liver puncture biopsy due to the risk of bleeding, with a Child–Pugh score of 5. The ICC for consistency of PHB measurements was 0.967 (95% CI: 0.872–0.992) in 10 PBC subjects by both observers. The ICC for the consistency of PHB measurements among the three observers with varying levels of seniority was found to be 0.943 (95% CI: 0.824–0.987) in the cohort of eight PBC subjects. Additionally, there were no statistically significant differences observed in the measured width of PHB between the three observers. The statistical, laboratory, and histological characteristics of the population in each group are shown in Table 1. The rate of missing data was less than 3%.

**Table 1:** Clinical and biological characteristics of subjects

Variable	PBC (*n* = 62)	HBV (*n* = 56)	*p* value
Age, y	51 (16–78)	47 (25–61)	0.009**
Women (*n*, %)	56 (90.3%)	36 (64.3%)	0.001***
BMI, kg/m^2^	22 (17–28)	22 (18–39)	0.591
ALT, U/L	27.0 (7.0–155.9)	25.0 (8.0–405.0)	0.781
AST, U/L	46.5 (13.4–210.0)	27.0 (10.0–412.0)	0.001***
TBIL, U/L	20.7 (7.5–204.7)	15.5 (3.0–72.5)	0.005**
ALP, U/L	171.5 (46.0–1100.0)	81.5 (38.0–209.0)	< 0.001****
GGT, U/L	96.5 (5.0–1637.0)	19.5 (6.0–715.0)	< 0.001****
PHB, mm	2.3 (0–6.7)	0.4 (0–4.1)	< 0.001****
Ludwig stage [*n* (%)]			
Stage 1	11 (18%)	-	
Stage 2	22 (35%)	-	
Stage 3	10 (16%)	-	
Stage 4	19 (31%)	-	
METAVIR score [*n* (%)]		-	
F1	-	17 (30%)	
F2	-	13 (23%)	
F3	-	12 (21%)	
F4	-	14 (25%)	

Data are shown as mean ± SD or median (range)

Abbreviations: *BMI*, body mass index; *ALT*, alanine aminotransferase; *AST*, aspartate aminotransferase; *TBIL*, total bilirubin; *ALP*, alkaline phosphatase; *GGT*, gamma-glutamyl transferase; *PHB*, periportal hypoechoic band; *PBC*, primary biliary cholangitis; *HBV*, hepatitis B virus. * *p* < 0.05; ** *p* < 0.01; *** *p* < 0.005; **** *p* < 0.001

### The correlation between PHB and histopathologic in PBC

**Fig. 2:**
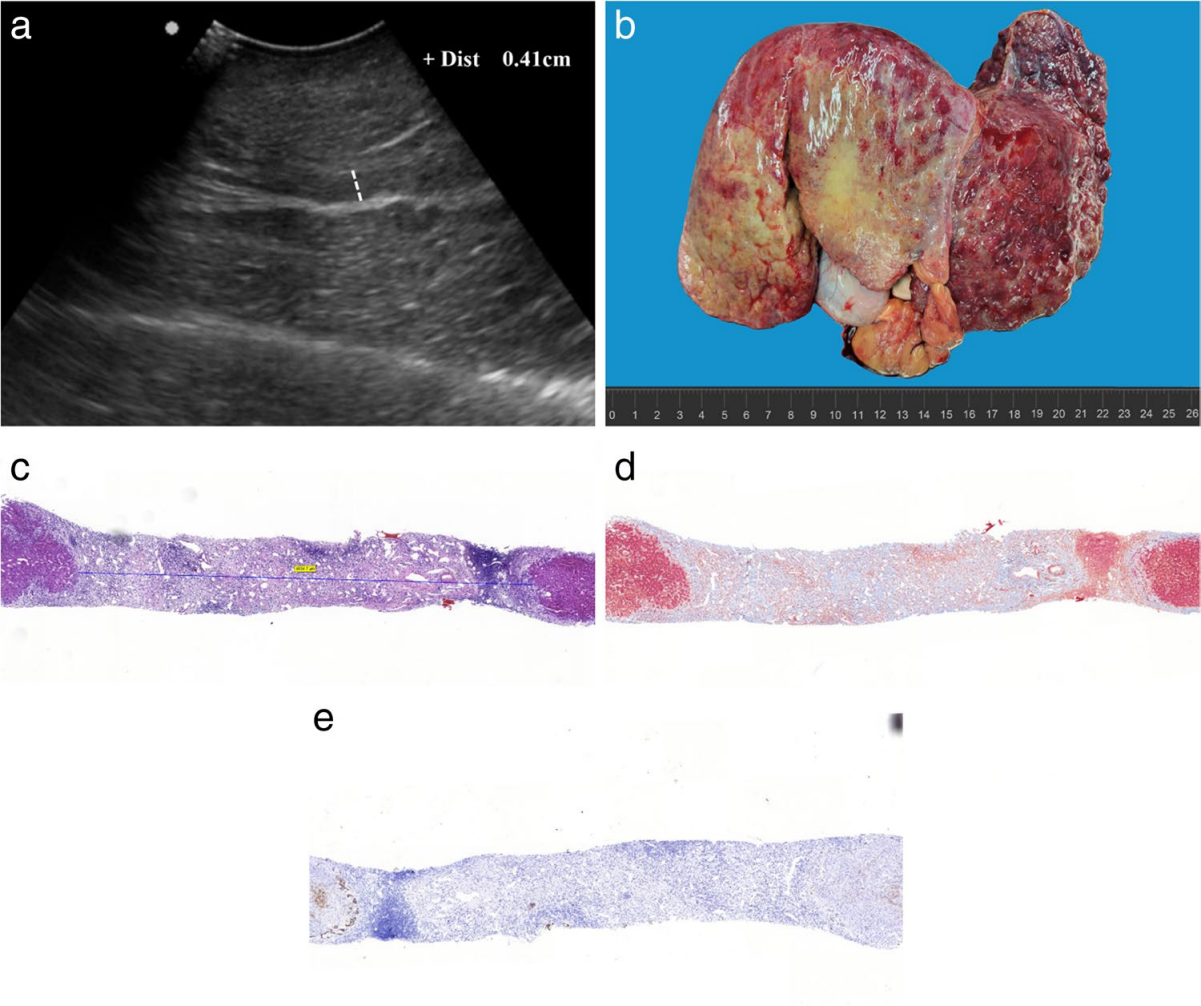
The ultrasound imaging and liver tissue biopsy of PHB around the portal vein of the diseased liver after transplantation. A stage 4 subject is a transplant patient with PBC, the measurement of PHB is 4.1 mm by ultrasound (**a**). **b** Liver specimens of a diseased liver after transplantation. **c** H/E stain shows many inflammatory cells in the PHB, the length of PHB was 4.1 mm. **d** Masson stain shows many fibroses in the PHB. **e** CK7 immunohistochemistry sections show few cholestasis in the PHB

Reviewing the histological specimens of PBC, we found that PHB was positively correlated with portal inflammation score and fibrosis score (*r* = 0.626, *r* = 0.666, all *p* < 0.001), but not with the degree of cholestasis (*p* = 0.172). One patient with stage 4 PBC was a liver transplant patient. The width of PHB measured by ultrasound was 4.1 mm. The liver tissue biopsy was performed on the PHB around the portal vein of the diseased liver after transplantation, and the puncture site was chosen to be the hypoechoic width around the rami inferior segment of the left external lobe of the portal vein. The biopsy results demonstrated that the PHB was composed of inflammatory cells infiltration, fibrous tissue hyperplasia, and a small amount of cholestasis, and the width of the area was 4.1 mm measured by multiheaded microscopy, which was consistent with the width measured by ultrasound (Fig. 2).

There was a strong correlation between the PHB and histological stage in patients with PBC (*r* = 0.844, *p* < 0.001), which widened with increasing stage. The median PHB of PBC patients was 1.4 (0–1.8) mm for stage 1, 2.1 (1.0–2.7) mm for stage 2, 3.0 (1.0–3.9) mm for stage 3, and 4.3 (2.9–6.7) mm for stage 4. The presence of PHB was 63.6% (7/11) in stage 1, and 100% in stage 2–stage 4. The degree of cholestasis in PBC with PHB was higher than those without the PHB group in stage 1 (*p* = 0.038).

### The diagnostic efficacy of PHB in PBC

**Fig. 3:**
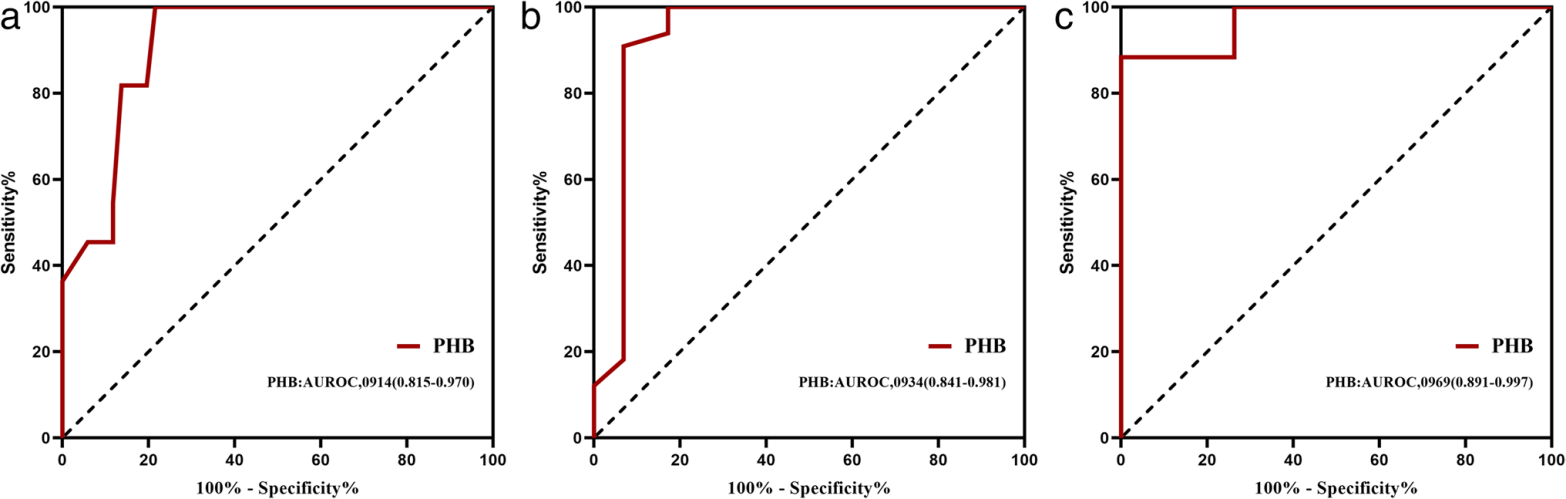
ROC curves for PHB measurement for different thresholds of the histological stage. Receiver operating characteristic (ROC) curve showing the prediction stage ≥ 2 (**a**), stage ≥ 3 (**b**), stage = 4 (**c**) with PHB in the PBC patient. In parentheses, 95% confidence intervals are shown

**Fig. 4:**
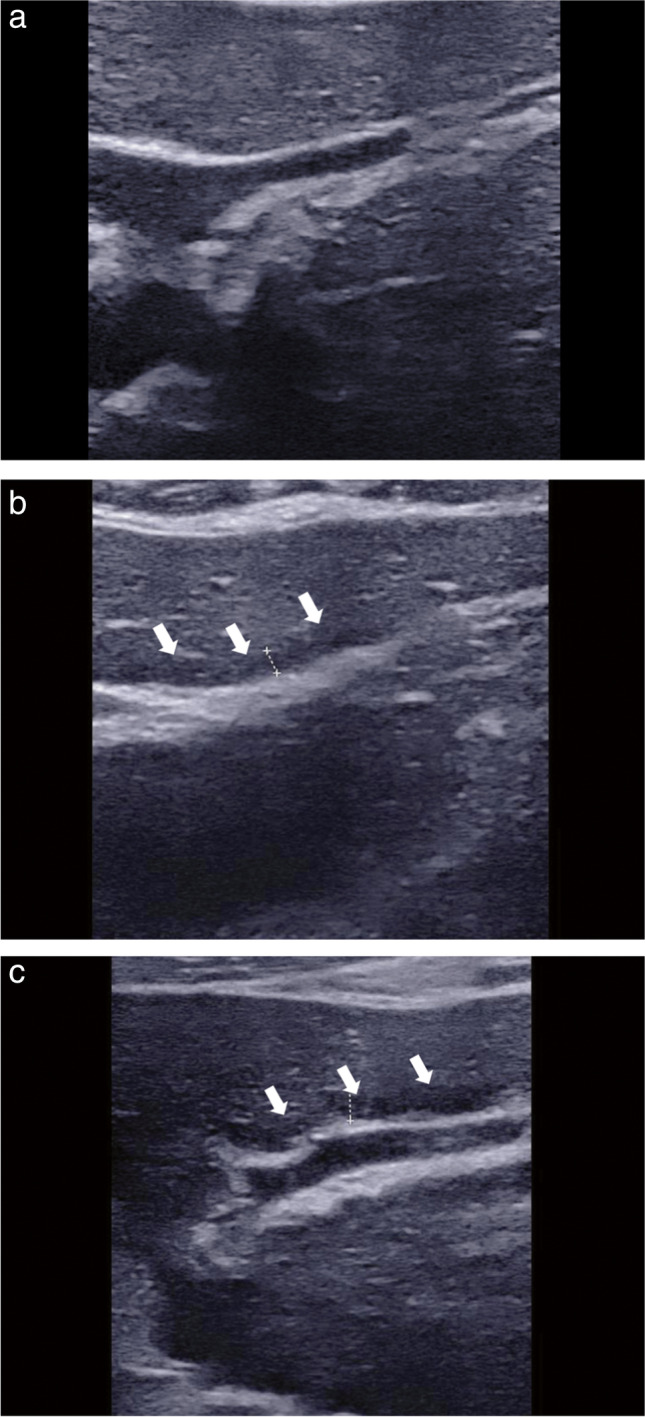
Comparison of echoes around the portal vein wall in PBC versus normal patients. Around the rami inferior segment of the left external lobe of the portal vein, the PHB was not found in normal patients (**a**); the PHB was characterized by uniform, regular, and evenly distributed in PBC patients (**b**, **c**). Abbreviation: PHB, periportal hypoechoic band

ROC curve analysis identified PHB > 1.8 mm (AUROC: 0.914; 95% CI: 0.815–0.970) as the best cutoff for predicting ≥ stage 2. For ≥ stage 3, the best cutoff PHB was 2.4 mm (AUROC: 0.934; 95% CI: 0.841–0.981). For = stage 4, the best cutoff PHB was 2.7 mm (AUROC: 0.969; 95% CI: 0.891–0.997) (Fig. 3, Table 2). when using 1.8 mm, 2.4 mm, and 2.7 mm cut-off values to identify the histological staging in PBC, 66.1% (41/62) were correctly predicted, and 21 were misclassified as following stage 1 for 18.2% (2/11), stage 2 for 54.5% (12/22), stage 3 for 70% (7/10), and stage 4 for 0% (0/19). It indicates that although this classification method has a high discriminatory ability, the accuracy of judgment is not satisfactory in practice. Figure 4 shows the comparison of echoes around the portal vein wall in PBC versus normal patients.

**Table 2:** Performance profile of PHB in differentiating stage

	Cutoff	AUROC (95% CI)	Sens, %	Spec, %	PPV (%)	NPV (%)	LR +	LR-
PHB in PBC patients (mm)								
Stage ≥ 2	1.8	0.914 (0.815–0.970)	78.4	100	100	50.0	-	0.22
Stage ≥ 3	2.4	0.934 (0.841–0.981)	93.1	90.9	90.0	93.7	10.24	0.076
Stage = 4	2.7	0.969 (0.891–0.997)	100.0	88.4	79.2	100	8.6	0.00
PHB in HBV patients (mm)								
F ≥ 2	1.0	0.729 (0.594–0.839)	56.4	88.2	91.7	46.9	4.79	0.49
F ≥ 3	1.0	0.735 (0.600–0.844)	65.4	76.7	70.8	71.9	2.8	0.45
F = 4	1.4	0.792 (0.662–0.889)	64.3	88.1	64.3	88.1	5.4	0.41

Abbreviations: *PHB*, periportal hypoechoic band; *PBC*, primary biliary cholangitis; *HBV*, hepatitis B virus

However, if the diagnostic threshold of PHB > 2.4 mm is used only to determine progressive (≥ stage 3) PBC, we are surprised to find that 90.3% (56/62) are correctly predicted and only 6 cases are misclassified, 4 of which are early and 2 of which are progressive. The level of AST and ALP were higher in 4 misclassified patients than in 29 correctly classified patients in the early stage of PBC [AST: 101.0 (70.0–136.0) vs 34.0(13.4–147.0) IU/L; ALP: 428.5 (176.0–1100.0) vs 171.0 (46.0–1100.0) IU/L], and the level of ALP and GGT were higher in 2 misclassified patients than in 27 correctly classified patients in the progressive stage of PBC [545.0 (490.0–600.0) vs 160.0 (81.0–653.0) IU/L; GGT: 545.0 (490.0–600.0) vs 160.0 (81.0–653.0) IU/L].

### Difference of PHB between PBC and HBV

To determine whether PHB is specific in PBC, we observed ultrasound images of 56 HBV patients and found that PHB was present in 50% of HBV. Comparing the differences in the degree of PHB, inflammation, and cholestasis between the two groups of subjects at the same fibrosis score level, we found that the PHBs were higher in PBC than in HBV in all subgroup; The degree of portal inflammation in the PBC group was higher than that in the HBV group in A subgroup and C subgroup and not significantly different from the HBV group in B subgroup; The degree of cholestasis was higher in the PBC group than in the HBV group in all subgroups (Table 3).

**Table 3:** The differences in portal inflammation, portal cholestasis, and PHB in different subgroups between PBC and HBV group

	A subgroup	B subgroup	C subgroup
	Median (range)	Median (range)	Median (range)
Scoring of portal inflammation			
PBC (*n* = 44)	1 (0–2)	2 (0–3)	3 (1–3)
HBV (*n* = 42)	0 (0–2)	1 (0–3)	1 (0–3)
* p* value	0.003***	0.101	0.03*
Scoring of portal cholestasis			
PBC (*n* = 44)	1 (0–3)	1 (1–3)	1 (1–3)
HBV (*n* = 42)	0 (0–2)	0 (0–1)	0 (0–2)
* p* value	0.001***	< 0.001****	0.002***
PHB (mm)			
PBC (*n* = 44)	1.5 (0–2.2)	2.2 (1.6–2.7)	3.5 (1–4.1)
HBV (*n* = 42)	0 (0–1.5)	0 (0–1.9)	1 (0–1.8)
* p* value	< 0.001****	< 0.001****	0.001***

Abbreviations: *PHB*, periportal hypoechoic band; *PBC*, primary biliary cholangitis; *HBV*, hepatitis B virus. * p < 0.05; **p < 0.01; *** p < 0.005; **** p < 0.001

In HBV patients, although there was a positive correlation between PHB and liver fibrosis stages (*r* = 0.514, *p* < 0.001), PHB did not better differentiate between ≥ F2 and ≥ F3 and had poor diagnostic efficacy in determining each liver fibrosis stage of HBV, (≥ F2, ≥ F3, = F4) AUROC of 0.729–0.792, sensitivity of 0.56–0.65 and specificity of 0.77–0.88 (Table 2).

### Univariate and multivariate regression analysis

To explore the risk factors influencing progressive PBC, PHB, age, sex, BMI, ALT, AST, TBIL, ALP, and GGT were included in a one-way regression analysis. The three variables with *p* < 0.05 in the univariate analysis including PHB, TBIL, and age were eventually included in the multifactorial logistic regression analysis, with only PHB odds ratio (OR) = 1.331, confidence interval (CI) = (1.105–1.603, *p* = 0.003) and TBIL (OR = 1.156, CI = 1.041–1.285, *p* = 0.007) ending up as independent influences on progressive PBC (Table 4).

**Table 4:** Univariate and multivariate analysis of risk factors associated with advanced-stage PBC patients

	Univariate Analysis		Multivariable Analysis	
Variable	OR (95%CI)	*p* value	OR (95%CI)	*p* value
Male gender	0.200 (0.022–1.823)	0.153	–	–
Age (years)	1.085 (1.028–1.145)	0.003	1.136 (0.999–1.293)	0.052
BMI (kg/m^2^)	1.017 (0.819–1.263)	0.880	–	–
ALT, U/L	0.990 (0.975–1.006)	0.214	–	–
AST, U/L	1.004 (0.993–1.017)	0.463	–	–
TBIL, U/L	1.079 (1.025–1.136)	0.004	1.156 (1.041–1.285)	0.007
ALP, U/L	0.999 (0.997–1.002)	0.640	–	–
GGT, U/L	1.000 (0.999–1.002)	0.947	–	–
PHB, × 10^2^ (mm)	1.280 (1.123–1.458)	< 0.001	1.331 (1.105–1.603)	0.003

Abbreviations: *BMI*, body mass index; *ALT*, alanine aminotransferase; *AST*, aspartate aminotransferase; *TBIL*, total bilirubin; *ALP*, alkaline phosphatase; *GGT*, gamma-glutamyl transferase; *PHB*, periportal hypoechoic band; *OR*, odds ratio; *CI*, confidence interval

## Discussion

We prospectively analyzed the diagnostic efficacy of PHB in the histological staging of the liver in patients with PBC. We found that PHB was better able to diagnose progressive PBC. PHB measurement is a non-invasive, simple, and reliable method for the assessment and follow-up of PBC disease progression.

Although the destruction and loss of intrahepatic bile ducts in PBC occur mainly in the small bile ducts, active inflammation can also be observed around the large bile ducts, which can be observed on radiologic imaging [10]. The periportal halo sign in MRI images reported in previous studies is [5], in our opinion, the PHB observed by ultrasound in this study. Initial case reports have suggested that a low-reflectivity periportal collar could be detected on hepatic ultrasound in two patients with PBC and one patient with primary sclerosing cholangitis (PSC), which was confirmed by hepatic puncture biopsy to be mononuclear cell inflammatory infiltrate with focal piecemeal necrosis [11]. Wenzel et al suggested that the periportal halo was due to fibrous tissue deposition or hepatocellular failure around the portal rather than by inflammation [6]. However, our study confirms that the PHB correlates not only with the degree of inflammatory infiltration in the portal area but equally with the degree of liver fibrosis. Malik et al state that the finding of the periportal halo sign is not limited to diseases with PBC, but is also present in diseases with autoimmune hepatitis (AIH) and overlap syndromes [12]. Combined with the typical pathological features of AIH, we consider inflammatory infiltrate of the portal and parenchyma interface one of the main causes of the formation of the periportal halo sign. The ultrasound manifestation of PHB in PSC patients has been reported in only one case report so far [11], and during our study, we also found the manifestation of PHB when reviewing the ultrasound images of PSC patients in our center. The PHB in PSC patients may be associated with the presence of inflammatory infiltration in the intra- and extrahepatic bile ducts and the pathological features of fibrosis in the portal area. To determine whether PHB is specific in autoimmune diseases, we matched HBV patients as controls. We found PHB in HBV patients as well, but the incidence and width of PHB were lower than in the PBC group. Analyzing the histological differences between the two diseases, the degree of inflammation in the portal area was less in HBV patients than in PBC in the early stages of liver fibrosis, and in the middle and late stages, the degree of cholestasis was increased in PBC patients. The difference in PHB between these two different liver diseases may be due to the two factors mentioned above.

In our study, PHB was not observed in four patients with PBC at histological stage 1. In the stage 1 cohort, the presence or absence of PHB correlated with the degree of cholestasis, but it has not been demonstrated that PHB widen with increasing cholestasis, and we consider that this may be influenced by the small sample size. In our study, the incidence of PHB in PBC was 93.5%, which was much higher than that reported in 43–66.7% [6, 13]. Wenzel et al and Kovač et al believed that the periportal halo sign occurs mainly during disease progression and its presence increases with increasing fibrosis [6, 13], which is consistent with our study, and we have the advantage of a relatively large study population. Our study has shown that the PHB in patients with early-stage PBC was characterized by a uniform, narrow, regular, and evenly distributed layer around the portal vein wall. Conversely, in patients with advanced PBC, the PHB was found to be a strip of varying width and narrow hypoechoic presentation. In this study, the visualization rate of the PHB by ultrasound was higher than that reported in previous MRI reports, which did not indicate that ultrasound had a stronger ability to visualize the PHB. We believe that in the early stage of the disease, mild inflammation and fibrosis in the small portal area will not affect the larger portal vein branches in the liver, and ultrasonography may show false positives in the PHB next to the larger portal vein branches. When examining the causes, it is possible that ultrasound waves reflect some of their energy when they encounter interfaces with significantly different acoustic impedances. This reflection may result in a low echo effect in front of the portal vein wall. It is suggested that this phenomenon could be attributed to lag-one coherence [14].

European Association for the Study of the Liver (EASL) clinical practice guidelines suggest that vibration-controlled transient elastography (VCTE) has been shown to be one of the best alternative markers for detecting cirrhosis or severe liver fibrosis in patients with PBC [3]. To date, previous studies on the diagnostic efficacy of TE for PBC are as follows: 0.744–0.92 AUROC for the determination of stage ≥ 2, 0.763–0.910 AUROC for ≥ 3, and 0.907–0.99 AUROC for = 4 [15–18]. In terms of the ROC for the determination of progressive fibrosis by TE, our study applies PHB for the determination of progressive PBC. The predictive power of PBC appears to be relatively high, other non-invasive assessment methods include shear-wave elastography (SWE) and real-time elastography (RTE) [16, 19], but study data are scarce and more data are needed to verify the diagnostic efficacy. Although our study found high AUROC for PHB to determine ≥ stage 2, ≥ stage 3, and = stage 4. Applying each diagnostic threshold to determine the histological staging of PBC is not recommended as it may increase misjudgment of stages 2 and 3, as well as lead to false-positive observations of PHB in the early stages of the disease. Although our diagnostic efficacy is not the highest among the various studies of non-invasive assessment, we have the advantage of a single disease type in the study population and a simpler method with relatively few influencing factors, which can be done quickly with plain 2D ultrasound.

In a multifactorial regression analysis, in addition to the traditional elevation of TBIL as a risk factor for disease progression in PBC [3], we found that widening of the PHB was an independent risk factor for progressive PBC. The widening of the PHB indicates increased liver fibrosis or increased inflammatory cells in the portal area and an increased risk of PBC disease progression. The study reveals that, although consistent results were obtained from observers of varying seniority in the measurement of PHB in 8 patients with PBC, it is necessary to validate the findings with a larger sample size. Furthermore, to standardize the measurement of PHB, it is essential to unify the measurement standards employed by different observers. Specifically, PHB measurements should be obtained in the middle of the inferior ramus segment of the left external lobe of the portal vein, the image should be magnified, and the results should be derived from the mean of multiple measurements. Although the application of PHB is convenient and quick to determine histological staging, HFUS measurements are affected by subcutaneous fat thickness and abdominal distension. Our study excluded 5 subjects who were unable to obtain HFUS measurements due to a BMI > 28 kg/m^2^ or abdominal distention. It can be seen that the staging of liver fibrosis by either the TE or HFUS is influenced by subcutaneous fat thickness and abdominal distention [4], but excluding these two causes, the role of PHB measurements in determining the degree of disease progression in patients with PBC cannot be underestimated.

There are some limitations of our study. First, due to the limitation of the low prevalence of PBC, we were unable to include a large sample size of PBC patients in a relatively short period of time. Moreover, because the patients with HBV who underwent liver biopsy during this study were predominantly young males, we were unable to include HBV patients whose age and sex were perfectly matched to those with PBC. Our study is single-center and we have not conducted further external verification at present. Therefore, we need to increase the sample size and conduct a multicenter study in the future to further explore in depth the factors influencing the histological staging of patients with PBC assessed by PHB. Secondly, to standardize the measurement criteria, cases limited by HFUS measurements were not applied to low-frequency ultrasound (LFUS) alternative measurements in our study, and in the future study, we intend to investigate whether LFUS combined with HFUS measurements can improve diagnostic accuracy.

In conclusion, the measurement of PHB provides a simple and easy assessment of the degree of disease progression in patients with PBC and provides an important clinical reference in predicting the histological staging of PBC from an ultrasound perspective.

## Abbreviations

AIH: Autoimmune hepatitis
ALP: Alkaline phosphatase
ALT: Alanine aminotransferase
AST: Aspartate aminotransferase
AUROC: Area under receiver operating characteristic curve
CI: Confidence interval
EASL: European Association for the Study of the Liver
GGT: Gamma-glutamyl transferase
HBV: Hepatitis B virus
HFUS: High-frequency ultrasound
ICC: Intraclass correlation efficient
LFUS: Low-frequency ultrasound.
MRI: Magnetic resonance imaging
OR: Odds ratio
PBC: Primary biliary cholangitis
PHB: Periportal hypoechoic band
PSC: Primary sclerosing cholangitis
ROC: Receiver operating characteristic
RTE: Real-time elastography
SWE: Shear-wave elastography
TBIL: Total bilirubin
TE: Transient elastography
VCTE: Vibration-controlled transient elastography

## Declarations

### Guarantor

The scientific guarantor of this publication is Yuan Zhang, MD.

### Statistics and biometry

One of the authors, Professor Xing Hu, has significant statistical expertise.

### Methodology

- prospective study
- performed at one institution
